# Risk Prediction for Non-alcoholic Fatty Liver Disease Based on Biochemical and Dietary Variables in a Chinese Han Population

**DOI:** 10.3389/fpubh.2020.00220

**Published:** 2020-07-02

**Authors:** Xinting Pan, Xiaoxu Xie, Hewei Peng, Xiaoling Cai, Huiquan Li, Qizhu Hong, Yunli Wu, Xu Lin, Shanghua Xu, Xian-e Peng

**Affiliations:** ^1^Fujian Provincial Key Laboratory of Environment Factors and Cancer, Department of Epidemiology and Health Statistics, School of Public Health, Fujian Medical University, Fuzhou, China; ^2^Key Laboratory of Ministry of Education for Gastrointestinal Cancer, Fujian Medical University, Fuzhou, China; ^3^Department of Cardiology, Affiliated Nanping First Hospital, Fujian Medical University, Nanping, China

**Keywords:** non-alcoholic fatty liver disease, non-invasive scoring method, nomogram, behavioral interventions, screening

## Abstract

Nonalcoholic fatty liver disease (NAFLD) is a common liver disease globally, but there are no optimal methods for its prediction or diagnosis. The present cross-sectional study proposes a non-invasive tool for NAFLD screening. The study included 2,446 individuals, of whom 574 were NAFLD patients. Multivariable logistic regression analysis was used to identify risk factors for NAFLD and incorporate them in a risk prediction nomogram model; the variables included both clinical and lifestyle-related variables. Following stepwise regression, BMI, waist circumference, serum triglyceride, high-density lipoprotein cholesterol, alanine aminotransferase, presence of diabetes and hyperuricemia, tuber and fried food consumption were identified as significant risk factors and used in the model. The final nomogram was found to have good discrimination ability (area under the receiver operating characteristic curve = 0.843 [95% *CI*: 0.819-0.867]), and reasonable accuracy for the prediction of NAFLD risk. A cut-off score of <180 for the nomogram was found to have high sensitivity and predictivity for the exclusion of individuals from screening. The model can be used as a non-invasive tool for mass screening.

## Introduction

Non-alcoholic fatty liver disease (NAFLD) is characterized by the presence of hepatic steatosis in the absence of secondary causes of hepatic accumulation such as excessive alcohol consumption (1), and it is associated with non-alcoholic steatohepatitis, hepatic fibrosis, liver cirrhosis and hepatocellular carcinoma (2–4). It is estimated that 25% of people worldwide and 21% of people in China suffer from NAFLD (5, 6). Further, liver-specific and all-cause mortality among NAFLD patients is 0.77 and 11.77 per 1,000 person-years (6), which makes it an enormous health burden. The causes are multifactorial and not completely understood, but NAFLD is reversible in the early stages. Although there is lack of pharmacological therapies, there are effective lifestyle interventions, such as energy restriction, dietary changes, and increase in physical activity (7, 8). These interventions are especially effective in the early stages of the disease. Therefore, methods for the early detection of NAFLD should be a key public health priority.

Currently, there are no published clinical markers for reliably predicting NAFLD. Liver biopsy remains the gold standard for diagnosis, but its drawbacks are its invasive nature, potential complications, and high cost (9, 10). On the contrary, ultrasonography is a non-invasive method that is more widely used for diagnosing NAFLD (11). However, ultrasonography, along with other imaging modalities such magnetic resonance imaging and computed tomography, is too expensive and inconvenient for routine health examinations and screening in a large population. Given these inherent limitations of liver biopsy and imaging modalities, in recent years, there has been increasing interest in the possibility of diagnosing NAFLD by using non-invasive clinical variables that are measurable in the peripheral blood (12–14). Thus, a few previous studies have focused on developing an NAFLD risk prediction model with non-invasive measures (15–18). The most commonly used variables in such models are biochemical indicators, including high-density lipoprotein cholesterol (HDL-c), total cholesterol (TC), and alanine transferase (ALT), but most predictive models contain one or two biomarkers that are not included in routine healthy examinations, such as serum insulin, hyaluronic acid, and α2-macroglobulin levels (19). Additionally, most of these indicators involve complicated calculation, and they do not take into consideration the impact of lifestyle characteristics, including dietary habits and the frequency of physical activities, which would be especially useful for assessing the effect of interventions (8). An ideal non-invasive test would be simple, easily available, low cost, and effective, and would provide easy visualization of the findings and identification of subjects at high risk of NAFLD. With such a test, large-scale population-wide screening and preventive programs in large populations would be possible. Therefore, the focus of this study was the development of optimal model for prediction and monitoring of NAFLD.

The aim of the present study was to use the known risk factors for NAFLD, such as age, obesity, lifestyle, and biochemical indicators, and develop an easily applicable prediction nomogram model for identifying subjects at high risk of NAFLD. A nomogram is a graphical depiction of prediction models that has been developed for a variety of clinical disease and tumors, but its application in NAFLD is rare. Therefore, the present nomogram might help in the construction of an early warning and prediction system for NAFLD that is suitable for the Chinese population.

## Materials and Methods

This cross-sectional study comprised 2,446 subjects who underwent abdominal ultrasonography between April 2015 and August 2017 at the Affiliated Nanping First Hospital of Fujian Medical University (Nanping, China). The study protocol conformed to the ethical guidelines of the 1,975 Declaration of Helsinki (6th revision, 2008) and was approved by the Ethics Committee of Fujian Medical University (ethical approval number 2014096). All the participants provided their informed consent before the study was started.

The inclusion criteria for participants in the current study were permanent residency in Nanping and age between 18 and 74 years and completed ultrasonography examination. The diagnosis of NAFLD in this cohort was primarily based on ultrasonographic findings rather than liver biopsy. This is because recent standardized criteria have significantly improved the diagnostic accuracy of ultrasonography so that even minor degrees of steatosis can be detected (20). Thus, NAFLD was diagnosed by liver ultrasonography, based on the following established criteria: [1] enhanced diffusion of the near-field echo and gradual attenuation of the far-field echo in the hepatic region, [2] unclear visual of the intrahepatic lacuna structure, [3] mild to moderate hepatomegaly with a round and blunt border, and [4] a reduction in the blood flow signal in the liver. The presence of criterion 1 and any one of criteria 2 to 4 is indicative of fatty liver (21).

The exclusion criteria were: [1] Abnormal energy intake (<600 kcal or >4,200 kcal per day for men; <500 kcal or >3,500 kcal per day for women), [2] alcohol abuse (weekly ethanol consumption: >140 g for men and >70 g for women) in the past year, and [3] a history of fatty liver or other liver diseases such as autoimmune hepatitis and drug-induced liver disease.

Data on traditional NAFLD risk factors were obtained through direct interviews with the help of a structured medical questionnaire. The risk factors included were age, sex, smoking, drinking, lifestyle, dietary habits, medical history, and family history of NAFLD. Subjects underwent a complete physical examination in the morning after an overnight fast. The clinical variables collected were height, weight, waist circumference, hip circumference, diastolic blood pressure and systolic blood pressure, serum triglyceride, TC, low-density lipoprotein cholesterol (LDL-c) and HDL-c, fasting plasma glucose, ALT, and aspartate aminotransferase (AST). All these variables were assessed using standard procedures. Body mass index (BMI) was calculated as body weight/(height)^2^. Continuous variables were grouped into four categories by quartiles.

**Figure 1:**
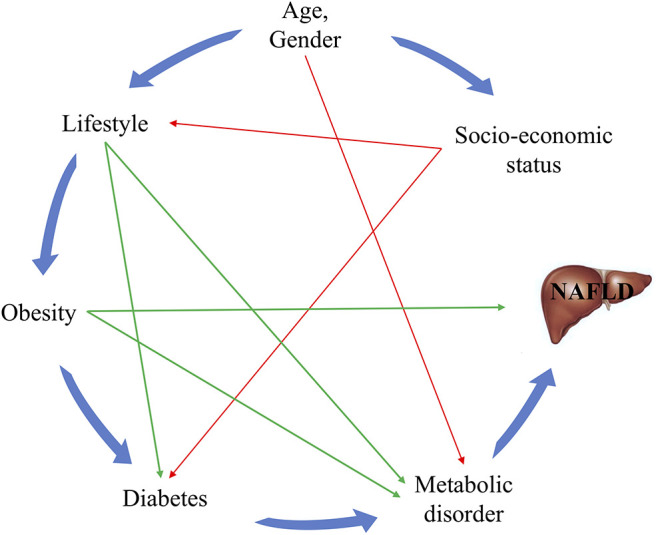
Directed acyclic graph for the relationships between known risk factors and NAFLD. The green lines represent the risk factors for which interventions can easily be provided, while the red lines indicate associations that are difficult to change.

Food consumption was assessed with the help of a food frequency questionnaire, and total consumption was calculated by multiplying the frequency of food consumption with the amount of food consumed each time. Hypertension was defined as systolic blood pressure ≥140 mm Hg and/or diastolic blood pressure ≥90 mm Hg, or current use of anti-hypertensive medication. Diabetes was defined as fasting plasma glucose ≥7.0 mmol/L, or current use of hypoglycemic agents. Hyperuricemia was defined as uric acid >420 μmol/L for men and >360 for women, or current use of hypouricemic agents. The possible relationships between these variables and NAFLD are depicted in the directed acyclic graph in Figure 1 (4).

**Figure 2:**
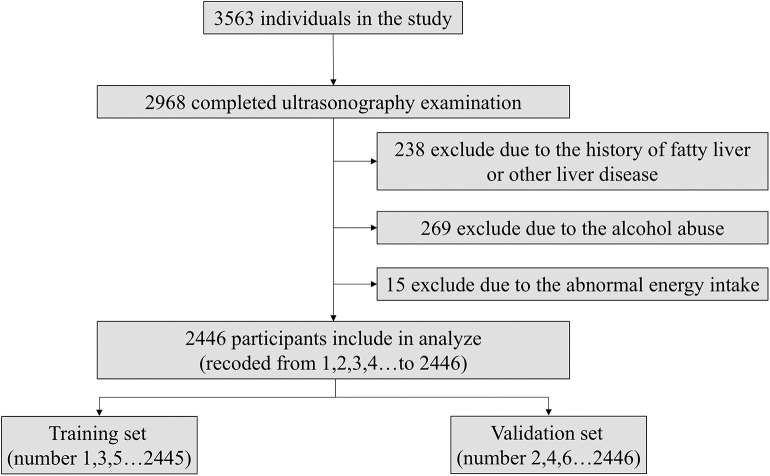
Flowchart of the study population.

As the flowchart shown in Figure 2, the subjects were numbered (from 1 to 2,446) according to the order in which they participated and divided into the training set (subjects with odd numbers like 1,3,5 et al.) and validation set (subjects with even numbers like 2,4,6 et al.). We used the model coefficients that were estimated in the training set for the discrimination and calibration analyses in the validation set. The sensitivity analysis about the ratio of training and validation set, sampling method were shown in Tables S1, S2.

### Statistical Analyses

All variables are presented by mean ± standard deviation, median (interquartile range), or counts with proportions, as indicated. Student's *t*-test or the non-parametric Mann-Whitney *U*-test, along with the chi-square test, were used to compare the variables between the NAFLD patients and healthy participants. Nomogram model involved a simple scoring system with which the probability of NAFLD could be determined. Multivariable logistic regression analysis was performed to estimate the odds ratio (*OR*) and 95% confidence interval (*CI*) and establish a nomogram using weighted estimators corresponding to each variable derived from fitted logistic regression coefficients, and forward stepwise variable selection was used to select the final model (22). The total score was calculated by summing the scores of each variable. A total point axis is obtained at the end of the nomogram and the probability of NAFLD was estimated by R software. The discriminatory ability of the nomogram was dependent on the area under the receiver operating characteristics (ROC) curve (AUC). Statistical analyses for nomogram construction, ROC and calibration curve were performed with the R software, and other statistical calculations were computed using SPSS, version 19.0.0.1 (IBM SPSS, 2010; Chicago, IL, USA). All *p*-values were two-tailed, and *p* < 0.05 was considered to indicate statistical significance.

## Results

Among the 2,446 participants, 574 were NAFLD patients. Thus, the prevalence of NAFLD was 23.5%. The subjects were divided into the training and validation sets for analysis. The demographic and clinical characteristics, and dietary habits are detailed in Table 1. Of the 1,223 individuals comprising the training set, 46.4% were male and the median (interquartile range) age was 43 (31–51) years. The median BMI and waist circumference were 22.31 kg/m^2^ and 80 cm, respectively. The majority of them were not smokers or drinkers, and the prevalence of diabetes, hypertension, hyperuricemia and NAFLD was 4.6, 17.7, 17.1, and 23.3%, respectively. In the validation set, the median age was 44 (31–52) years, and 49.6% were male. The characteristics of both sets were similar.

**Table 1:** Baseline characteristics of the participants in the two sets.

**Characteristics**	**Training set (*n* = 1,223)**	**Validation set (*n* = 1,223)**	***P*-value**
**Demographic characteristics**			
Male (*n*, %)	568 (46.4%)	606 (49.6%)	0.124
Age (y)	43 (31–51)	44 (31–52)	0.137
BMI (kg/m^2^)	22.31 (20.55–24.51)	22.49 (20.55–24.61)	0.540
Waist circumference (cm)	80 (74–86)	80 (74–87)	0.650
Smoker (*n*, %)	243 (19.9)	266 (21.7)	0.252
Drinker (*n*, %)	331 (27.1)	313 (25.6)	0.409
Diabetes (*n*, %)	56 (4.6)	61 (5.0)	0.636
Hypertension (*n*, %)	217 (17.7)	220 (18.0)	0.874
Hyperuricemia (*n*, %)	209 (17.1)	200 (16.4)	0.626
NAFLD (*n*, %)	285 (23.3)	289 (23.6)	0.849
**Clinical characteristics**			
TC (mmol/L)	4.98 (4.54–5.49)	4.97 (4.48–5.46)	0.452
Triglyceride (mmol/L)	1.14 (0.88–1.61)	1.15 (0.88–1.64)	0.884
HDL-c (mmol/L)	1.36 (1.18–1.47)	1.35 (1.15–1.46)	0.140
LDL-c (mmol/L)	3.07 (2.66–3.55)	3.11 (2.66–3.52)	0.931
ALT (IU/L)	18 (13–26)	18 (14–26)	0.375
AST (IU/L)	21 (18–24)	21 (18–25)	0.461
**Dietary habits**			
Fried foods (g/week)	45.5 (24.5–90.3)	45.5 (24.5–90.7)	0.972
Tuber (g/week)	102.9 (36.8–147.0)	102.9 (36.7–147)	0.699
Dairy products (g/day)	52.5 (17.5–250.0)	52.5 (17.5–250.0)	0.869
Meat and poultry (g/day)	73.7 (46.0–117.0)	72.8 (45.2–113.0)	0.553
Fish (g/day)	25.0 (14.0–49.0)	24.0 (13.2–43.1)	0.454

*BMI, body mass index; NAFLD, nonalcoholic fatty liver disease; TC, total cholesterol; HDL-c, high-density lipoprotein cholesterol; LDL-c, low-density lipoprotein cholesterol; ALT, alanine aminotransferase; AST, aspartate aminotransferase*.

**Figure 3:**
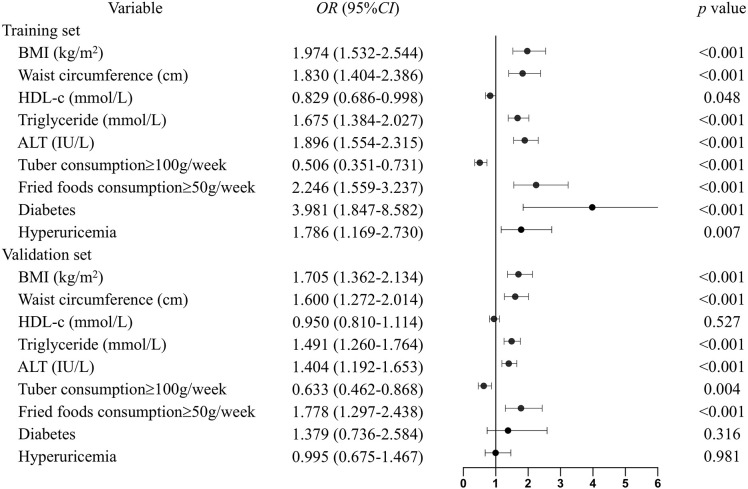
Multivariable odd ratios for the relationship between the eight identified risk factors and NAFLD. Continuous variables were subsequently classified by quartiles, and the first quartile was used as the reference category. The following scores were assigned to the quartiles for BMI: 1, <20.5; 2, 20.5–22.4; 3, 22.4–24.6; and 4, >24.6 kg/m^2^. Waist circumference: 1, <74; 2, 74–80; 3, 80–87; and 4, >87 cm. HDL-c: 1, <1.17; 2, 1.17–1.36; 3, 1.36–1.46; and 4, >1.46 mmol/L. Triglyceride: 1, <0.88; 2, 0.88–1.15; 3, 1.15–1.62; and 4, >1.62 mmol/L. ALT: 1, <13; 2, 13–18; 3, 18–26; and 4, >26 IU/L. The groups were subdivided into subgroups based on tuber and fried food consumption, based on cutoff points of 100 g/weeks and 50 g/weeks, respectively. For diabetes and hyperuricemia: 1 means the presence of the diseases and 0 means not.

All the recorded variables were examined for association with NAFLD, and multivariate odd ratios were calculated to build the nomogram (Figure 3). In the training set, elevated BMI (*OR* = 1.974, *CI* = 1.532–2.544), waist circumference (*OR* = 1.830, *CI* = 1.404–2.386), serum triglyceride (*OR* = 1.675, *CI* = 1.384–2.027), ALT (*OR* = 1.896, *CI* = 1.554–2.315), fried food consumption (*OR* = 2.246, *CI* = 1.559–3.237), and the presence of diabetes (*OR* = 3.981, *CI* = 1.847–8.582), and hyperuricemia (*OR* = 1.786, *CI* = 1.169–2.730) were associated with a higher risk of NAFLD, while higher serum HDL-c (*OR* = 0.829, *CI* = 0.686–0.998) and tuber consumption (*OR* = 0.506, *CI* = 0.351–0.731) were associated with a lower risk of the disease. These relationships were similar in the validation set, except the serum HDL-c and the presence of diabetes and hyperuricemia.

**Figure 4:**
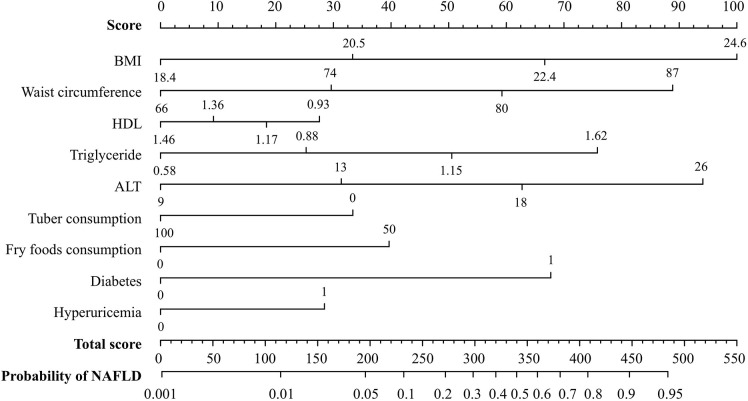
A nomogram for predicting the risk of NAFLD. A line is drawn from the point corresponding to the value of each variable on its axis to the score scale on top. The scores for each variable are summed up to determine the total score. A line is drawn from the total score on the total score axis to the bottom axis depicting the probability of NAFLD in order to determine the probability of NAFLD.

The final nomogram that includes all eight variables can be used to estimate the risk of NAFLD. The weighted estimators and score of each variable were shown in Table S3. Each predictive variable is scored by locating its position on its scale and drawing a straight line to the scoring scale on top. The scores for each variable are summed up to calculate the total score. The total score scale and NAFLD probability scale are located at the bottom, and a vertical line is drawn from the point representing the total score to the scale showing the probability of NAFLD (Figure 4). For example, a person who is diabetes patient would fall under category 1 (score = 68 on the scoring scale on top). If his BMI, waist circumference, serum HDL-c, TG, and ALT levels are 24.5 kg/m^2^ (category 3, score = 67), 85 cm (category 3, score = 59), 0.91 mmol/L (category 1, score = 28), 3.01 mmol/L (category 4, score = 76), and 28 U/L (category 4, score = 94), respectively, and he consume 75 g of tubers (category 0, score = 33) and 100 g of fried foods (category 1, score = 40) per week, the total risk score is 465 points, which corresponds to an 93% probability of NAFLD, in accordance with the probably scale at the bottom.

**Figure 5:**
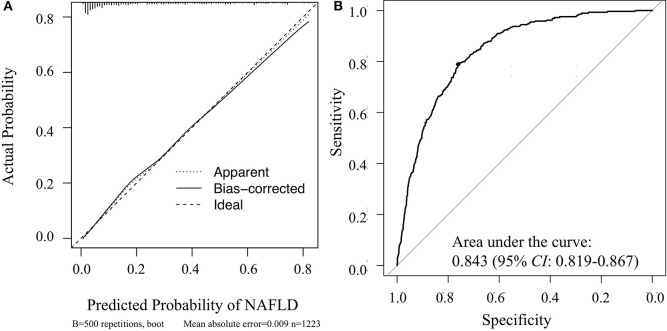
Veritified the nomogram for determining the risk of NAFLD in the validation set. **(A)** Calibration curve of the nomogram; **(B)** receiver operating characteristic curve of the nomogram.

Hosmer-Lemeshow goodness-of-fit tests yielded chi-square values of 4.582 (*p* = 0.801) and 10.002 (*p* = 0.265) for the training and validation sets, respectively. These values indicate that the deviation between the predicted and observed events in the two datasets was not significant. A calibration curve drawn showed that the predicted probabilities reasonably approximated the actual prevalence of NAFLD in the validation set; thus, the nomogram provided good calibration (Figure 5A). In addition, the model was validated and demonstrated good discrimination and with AUC of 0.843 (95% *CI*: 0.819–0.867) for the validation set (Figure 5B).

Table 2 gives the sensitivity, specificity, positive predictive value and negative predictive value for 40-unit intervals of the score. The cut-off points 180 and 340 divided participants into 2:2:1. The sensitivity analysis about cut-off points in training set were shown in Table S4. In the training set, the median scores are 371 (322–418) and 180 (107–259) for NAFLD cases and healthy participants, respectively. As shown in Table 3, NAFLD can be ruled out when the total score is less than 180, with a sensitivity of 98.60% (95%*CI*: 97.22–99.97%) and a negative predictive value of 99.15% (95%*CI*: 98.32–99.98%). When the total score is more than 340, NAFLD can be predicted with a specificity of 93.71% (95% *CI*: 92.15–95.27%) and a positive predictive value of 75.92% (95% *CI*: 70.53–81.31%). Based on these two cut-off points of <180 and >340, each of the groups were classified into three subgroups: 472 (38.6%) individuals had scores of <180, 245 (20.0%) had scores of >340, and 506 (41.4%) had scores of 180–340, with the percentage of NAFLD patients in these three groups being 0.8, 18.8, and 75.9%, respectively. Participants with scores of >340 and scores of 180–340 had a higher risk of NAFLD than those with scores of <180 (both *p* < 0.001).

**Table 2:** Diagnostic accuracy of the score.

**Score cut-off point**	**%**	**SN (%)**	**SP (%)**	**PPV (%)**	**NPV (%)**
**Training set**					
≥140	72.2	99.6	36.1	32.2	99.7
**≥180**	**61.4**	**98.6**	**49.9**	**37.4**	**99.2**
≥220	51.3	95.4	62.0	43.3	97.8
≥260	39.9	90.2	75.4	52.7	96.2
≥300	29.0	79.6	86.4	63.9	93.3
**≥340**	**20.0**	**65.3**	**93.7**	**75.9**	**89.9**
≥380	12.4	44.2	97.2	82.9	85.2
**Validation set**					
≥140	71.7	97.6	36.3	32.2	98.0
≥180	62.7	95.5	47.4	36.0	97.1
≥220	51.7	91.0	60.5	41.6	95.6
≥260	42.1	83.0	70.6	46.6	93.1
≥300	32.2	70.9	79.8	52.0	89.9
≥340	21.2	54.7	89.2	61.0	86.4
≥380	13.4	36.7	93.8	64.6	82.7

*%, number of participants with score≥ cut-off point; SN, sensitivity; SP, specificity; PPV, positive predictive value; NPV, negative predictive value*.

**Table 3:** Association between nomogram scores and NAFLD.

	**Low risk (score <180)**	**Moderate (score 180–340)**	**High risk (score > 340)**
**Training set**			
Median (interquartile range)	107 (69–145)	256 (221–295)	392 (364–427)
Total, *n* (%)	472 (38.6)	506 (41.4)	245 (20.0)
NAFLD, *n* (%)	4 (0.8)	95 (18.8)	186 (75.9)
Sensitivity (%)	98.60 (97.22–99.97)		65.26 (59.70–70.82)
Specificity (%)	49.89 (46.69–53.10)		93.71 (92.15–95.27)
Positive predictive value (%)	-		75.92 (70.53–81.31)
Negative predictive value (%)	99.15 (98.32–99.98)		-
**Validation set**			
Median (interquartile range)	103 (67–138)	260 (217–302)	389 (369–422)
Total, *n* (%)	456 (37.3)	508 (41.5)	259 (21.2)
NAFLD, *n* (%)	13 (2.9)	118 (23.2)	158 (61.0)
Sensitivity (%)	95.50 (93.10–97.91)		54.67 (48.90–60.44)
Specificity (%)	47.43 (44.22–50.64)		89.19 (87.19–91.18)
Positive predictive value (%)	-		61.00 (55.02–66.98)
Negative predictive value (%)	97.15 (95.62–98.68)		-

In the validation set, the median scores were 350 (292–402) and 189 (104–274) for NAFLD cases and other subjects, respectively. At the cut-off value of <180, NAFLD could be ruled out with a sensitivity of 95.50% (95% CI: 93.10–97.91%) and a negative predictive value of 97.15% (95% CI: 95.62–98.68%). At the cut-off value of >340, NAFLD could be detected with a specificity of 89.19% (95% *CI*: 87.19–91.18%) and a positive predictive value of 61.00% (95%*CI*: 55.02–66.98%). There were 456 (37.3%) individuals with a score that was <180, 259 individuals (21.2%) with a score that was >340, and 508 individuals (41.5%) with a score of 180–340. The incidence of NAFLD in the <180, 180–340, and >340 groups was 2.9, 23.2, and 61.0%, respectively. A score of >340 and 180–340 was associated with a higher risk of NAFLD than a score of <180 (both *p* < 0.001).

**Figure 6:**
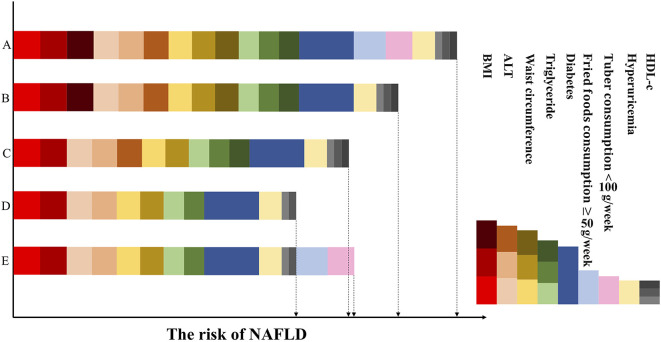
Dynamic risk score based on changes in obesity, dietary, clinical variables and related diseases. The figure represents the same patient over the intervention period. In the first column, row A represents the patient's score at which point he has unfavorable BMI and waist circumference, high serum triglyceride levels, low HDL-c levels, and impaired ALT levels, developed diabetes and hyperuricemia, and eats little tuber and a lot of fried foods. The risk decreases in row B, as consumption of tuber increased and restriction on fried foods (intervention strategy 1). Row C reflects the efforts of the patient to stay lean and maintain his waist circumference at <87 cm (intervention strategy 2): the risk score further decreases. Row D shows the patient has relatively normal serum HDL-c, triglyceride and ALT levels on account of the previous intervention strategies and has a low risk of NAFLD. While in the final one, row E, at which point his clinical characteristics are unchanged but he had bad dietary habits again. As a result, his risk score has increased and higher than row D. Red, brown, dark red means BMI: 20.5–22.4, 22.4–24.6, and >24.6 kg/m; Peach puff, sandy brown, chocolate means serum ALT: 13–18, 18–26, and >26 IU/L; Gold, goldenrod, dark khaki means waist circumference: 74–80, 80–87, and >87 cm; Light green, green, dark green means triglyceride: 0.88–1.15, 1.15–1.62, and >1.62 mmol/L; Dark blue means the presence of diabetes; Light blue means the consumption of fried foods ≥50 g/weeks; Pink means the consumption of tuber <100 g/weeks; Wheat means the presence of hyperuricemia; Light gray, gray, dark gray means HDL-c: 1.36–1.46, 1.17–1.36m and <1.17 mmol/L.

Besides, a patient can be scored over successive timepoints in order to assess the effect of interventions on the risk of NAFLD, as shown in the example in Figure 6.

## Discussion

In the present cross-sectional study, we developed a nomogram model for predicting NAFLD and validated it internally. The model showed high sensitivity and predictivity for determining the risk of NAFLD. The nomogram includes eight variables associated with the risk of NAFLD: BMI, waist circumference, serum triglyceride, HDL-c, ALT, tuber and fried food consumption, and the presence of diabetes and hyperuricemia. It is easy to collect data for these variables, as well as monitor changes in their values. Thus, this nomogram model may be a valuable tool for assessing the effect of lifestyle interventions on the risk of NAFLD.

Considered as a hepatic manifestation of metabolic syndrome, NAFLD is associated with obesity, type 2 diabetes, dyslipidemia and hyperuricemia (4, 23–25). Consistent with the findings of previous studies, we found that high BMI, waist circumference, and serum triglyceride levels were risk factors for NAFLD, and high HDL-c level was a protective factor. Weight loss can have important benefits in NAFLD patients, and several potential mechanisms have been suggested to explain these benefits. One of these mechanisms is a reduction in hepatic steatosis and liver enzyme levels. Another proposed mechanism is that weight loss can decrease the risk of cardiovascular disease and diabetes and further reduce the risk of NAFLD (26, 27). Thus, the previous and present findings, in general, imply that weight loss interventions may be highly beneficial for reducing the risk of NAFLD or alleviating NAFLD. In the current study, unhealthy diet was also found to be a risk factor for NAFLD. Overconsumption of fried foods is associated with an increase in the intake of calories, saturated fatty acids, and cholesterol, and this in turn, may lead to an increase in hepatic lipid storage and insulin resistance (28, 29). On the other hand, tubers contain high amounts of starch and antioxidants, which are known to have beneficial effects on health. Furthermore, potato starch provides large amounts of volatile fatty acids during the post-absorptive period, and this can regulate liver lipid metabolism (30). In this regard, Hossein et al. reported that the consumption of boiled potato can eliminate the risk of diabetes because of the remarkably high fiber content (31). As reported previously (32, 33), excessive intake of fried foods and low intake of tuber were risk factors for NAFLD in this study. Therefore, the general consensus from the reported studies, as well as the present findings, is that dietary interventions can also reduce the risk of NAFLD or improve already present NAFLD.

BMI, waist circumference and serum triglyceride, ALT, AST were widely used in NAFLD risk scoring models. For example, NAFLD liver fat score including the presence of the metabolic syndrome and type 2 diabetes, fasting serum insulin, AST, and the AST/ALT ratio, had an AUC of 0.86 in the validation group (34). Regrettably, we did not obtain serum insulin, the model cannot used for evaluation in current study. Italian researchers combined BMI, waist circumference, serum γ-glutamyl transpeptidase and triglyceride to develop the fatty liver index (16). However, when used for our population, the AUC in the training and validation sets were 0.893 (lower than 0.913 of our model, *p* = 0.002) and 0.835 (*p* = 0.738), respectively. The result showed that the fatty liver index may not suitable for Chinese population who have lower BMI and waist circumference than the Italian. In another study (35), researchers collected 509 patients with MetS and developed a risk scoring scheme which including BMI≥25, AST/ALT≥1, ALT≥40, type 2 diabetes mellitus, and central obesity. When applied in validation cohort, the positive predictive value of NAFLD in patients with low-risk (scores below 3), and negative predictive value of high-risk (scores 5 and over) were 84 and 100%, respectively.

The AUC for the present nomogram model was 0.843 (95% *CI*: 0.819–0.867). Although this is far from optimal, the value does confirm that this model can be utilized to select eligible individuals for ultrasonography-based screening and is likely to improve the cost-effectiveness of screening. Further, the lower cut-off scores of <180 showed high sensitivity and negative predictive value in both the study sets. This means that individuals with scores of <180 can be excluded from NAFLD screening, and the associated costs to the patient as well as healthcare system can be eliminated. In the present study, 38.6 and 37.3% of the individuals from the training and validation set, respectively, had scores of <180. This means that ultrasonography was not deemed necessary in 40% of these subjects, based on the present model. However, it should be noted that the positive predictive value of the upper cut-off point (340) was not very high. Despite this, we believe that the nomogram will be a useful prescreening test for NAFLD in the Chinese Han population.

An appealing aspect of our model is its dynamic nature, as the lifestyle variables and biomarkers included tend to change over time or in response to interventions. For example, BMI, waist circumference, and dietary habits are modifiable; related diseases and clinical indicators such as serum TG, HDL-c, and ALT can be altered based on other lifestyle characteristics. For example, as shown in Figure 6, the risk score in row B is reduced because of the healthy diet as compared with A; and the risk score in row C is also reduced as the result of decrease in BMI and waist circumference as compared with B. While the risk score in row E is increased because of the unhealthy dietary habits, despite the decrease in clinical characteristics as compared with row C. This indicates that the modifiable factors are in a pre-eminent position in the process of NAFLD. With regard to the inter-relationships between these factors in the context of NAFLD, it is known that an unhealthy lifestyle can result in obesity, diabetes and dyslipidemia, and that obesity and diabetes also lead to dyslipidemia (Figure 1). Therefore, it follows that making lifestyle changes such as consuming a healthier diet and engaging in more physical activity can gradually result in a decrease in the risk of NAFLD or alleviate already existing NAFLD (8, 36), as depicted in the case represented in Figure 6.

One of the advantages of this model over previous ones is that it allows for better visualization of risk prediction, and therefore, allows for easy screening of high-risk groups. Another related advantage is that it can improve the cost-effectiveness of screening by eliminating low-risk individuals. Finally, as the model contains modifiable factors, monitoring the scores over time may be useful for understanding the effects of prevention strategies for NAFLD. This study also has some potential limitations. First, the intake of tuber and fried food were assessed by food frequency questionnaire, and the amount of consumption was self-reported. There has an inherent limitation of recall bias, participants may over-report or under-report the consumption of foods. While, all subjects were investigated the dietary habits 1 year prior to interview and all NAFLD cases were new diagnosed, which might minimize the recall bias. Second, lifestyle and average clinical indicators may vary across different ethnic groups, and the current study includes the Chinese Han population only, the model presented here may not be reasonably applied to the general population or other ethnicities. Therefore, this nomogram needs to be modified and reassessed the cut-off points in other populations before it can be widely applied. Third, although the nomogram showed high performances in internal validation, it still requires further external validation to verify our results, especially those including different ethnic groups.

## Conclusion

Based on the findings of previous investigations, we identified a set of demographic, clinical, and dietary variables that was used to build a nomogram model for predicting the risk of NAFLD. The nomogram developed in this study was internally validated for predicting NAFLD in Chinese Han adults, and it has potential as a non-invasive and cost-effective method to screen for individuals with high NAFLD risk and to assess the effects of lifestyle interventions, such as dietary improvements and increased levels of physical activity. Furthermore, it can provide support for targeted health education and behavioral interventions. However, this model needs to be validated externally and modified for other population cohorts before it can be widely applied.

## Data Availability Statement

The datasets generated for this study are available on request to the corresponding author.

## Ethics Statement

The studies involving human participants were reviewed and approved by the Ethics Committee of Fujian Medical University. The patients/participants provided their written informed consent to participate in this study.

## Author Contributions

SX and XPe contributed conception and design of the study. XPa, HP, XC, HL, and QH collected investigation and organized the database. XPa performed the statistical analysis and wrote the first draft of the manuscript. XX, YW, XL, and XPe contributed to manuscript revision. All authors read and approved the submitted version.

## Conflict of Interest

The authors declare that the research was conducted in the absence of any commercial or financial relationships that could be construed as a potential conflict of interest.
