# Supplementary material for: Risk Prediction for Non-alcoholic Fatty Liver Disease Based on Biochemical and Dietary Variables in a Chinese Han Population
Source: Front Public Health. 2020 Jul 2;8:220. doi: 10.3389/fpubh.2020.00220 (PMC7346601; doi:10.3389/fpubh.2020.00220)
Supplement: Supplementary file 1 [file Table_1.DOCX]

Table S1. Sensitivity analysis about the ratio of training and validation set

| **Model** | **The ratio of training and validation set** | **The number of variables** | **The AUC of new model in validation group** | **The AUC of model 1 in validation group** | ***P* value** |
| --- | --- | --- | --- | --- | --- |
| Model 1 | 1:1 | 9 | 0.843(0.819-0.867) | 0.843(0.819-0.867) | - |
| Model 2 | 2:1 | 12 | 0.856(0.828-0.885) | 0.865(0.836-0.893) | 0.097 |
| Model 3 | 3:1 | 11 | 0.856(0.823-0.888) | 0.849(0.815-0.883) | 0.064 |
| Model 4 | 7:3 | 13 | 0.863(0.833-0.892) | 0.862(0.832-0.892) | 0.891 |
