# Supplementary material for: Risk Prediction for Non-alcoholic Fatty Liver Disease Based on Biochemical and Dietary Variables in a Chinese Han Population
Source: Front Public Health. 2020 Jul 2;8:220. doi: 10.3389/fpubh.2020.00220 (PMC7346601; doi:10.3389/fpubh.2020.00220)
Supplement: Supplementary file 2 [file Table_2.DOCX]

Table S2. Sensitivity analysis about sampling method

| **Sampling method** | **Model** | **The number of variables** | **The AUC of new model in validation group** | **The AUC of model 1 in validation group** | ***P* value** |
| --- | --- | --- | --- | --- | --- |
| Systematic sampling | Model 1 | 9 | 0.843(0.819-0.867) | 0.843(0.819-0.867) | - |
| Random sampling 1 | Model 2 | 10 | 0.860(0.838-0.883) | 0.871(0.849-0.892) | 0.018 |
| Random sampling 2 | Model 3 | 10 | 0.876(0.854-0.897) | 0.876(0.854-0.898) | 0.942 |
| Random sampling 3 | Model 4 | 9 | 0.881(0.860-0.901) | 0.886(0.866-0.906) | 0.186 |
