# Supplementary material for: Risk Prediction for Non-alcoholic Fatty Liver Disease Based on Biochemical and Dietary Variables in a Chinese Han Population
Source: Front Public Health. 2020 Jul 2;8:220. doi: 10.3389/fpubh.2020.00220 (PMC7346601; doi:10.3389/fpubh.2020.00220)
Supplement: Supplementary file 3 [file Table_3.DOCX]

Table S3. The weighted estimators and score of each variable

| **Variables** | **β value** | **Groups** | **Weighted estimators** | **Scores** |
| --- | --- | --- | --- | --- |
| BMI | 0.68 | 1,2,3,4 | 2.04 | 0/33/67/100 |
| ALT | 0.64 | 1,2,3,4 | 1.92 | 0/31/63/94 |
| Waist circumference | 0.604 | 1,2,3,4 | 1.812 | 0/30/59/89 |
| TG | 0.516 | 1,2,3,4 | 1.548 | 0/25/51/76 |
| Diabetes | 1.382 | 0,1 | 1.382 | 0/68 |
| Consumption of fry foods | 0.809 | 0,1 | 0.809 | 0/40 |
| Consumption of tuber | -0.681 | 0,1 | -0.681 | 33/0 |
| Hyperuricemia | 0.58 | 0,1 | 0.58 | 0/28 |
| HDL | -0.188 | 1,2,3,4 | -0.564 | 28/19/9/0 |
