# Supplementary material for: Risk Prediction for Non-alcoholic Fatty Liver Disease Based on Biochemical and Dietary Variables in a Chinese Han Population
Source: Front Public Health. 2020 Jul 2;8:220. doi: 10.3389/fpubh.2020.00220 (PMC7346601; doi:10.3389/fpubh.2020.00220)
Supplement: Supplementary file 4 [file Table_4.DOCX]

Table S4. Sensitivity analysis about cut-off points in training set

| **Low cut-off point** | **High cut-off point** | **SN (%)** | **NPV (%)** | **SP (%)** | **PPV (%)** | **SN+SP-1** | **Subjects<low cut-off point or >high cut-off point (%)** | **Correctly classified (%) *** |
| --- | --- | --- | --- | --- | --- | --- | --- | --- |
| 140 | 300 | 99.6 | 99.7 | 86.4 | 63.9 | 0.86 | 56.83 | 46.28 |
| 140 | 340 | 99.6 | 99.7 | 93.7 | 75.9 | 0.93 | 47.83 | 42.93 |
| 140 | 380 | 99.6 | 99.7 | 97.2 | 82.9 | 0.97 | 40.23 | 38.02 |
| 180 | 300 | 98.6 | 99.2 | 86.4 | 63.9 | 0.85 | 67.62 | 56.83 |
| 180 | 340 | 98.6 | 99.2 | 93.7 | 75.9 | 0.92 | 58.63 | 53.48 |
| 180 | 380 | 98.6 | 99.2 | 97.2 | 82.9 | 0.96 | 51.02 | 48.57 |
| 220 | 300 | 95.4 | 97.8 | 86.4 | 63.9 | 0.82 | 77.68 | 66.15 |
| 220 | 340 | 95.4 | 97.8 | 93.7 | 75.9 | 0.89 | 68.68 | 62.80 |
| 220 | 380 | 95.4 | 97.8 | 97.2 | 82.9 | 0.93 | 61.08 | 57.89 |

SN, sensitivity of low cut-off point; NPV, negative predictive value of low cut-off point; SP, specificity of high cut-off point; PPV, positive predictive value of high cut-off point; *: the percentage of correctly classified participants in all subjects of training set.
